# Impact of guselkumab on three cases of SSc accompanying psoriasis

**DOI:** 10.1093/rheumatology/kead287

**Published:** 2023-06-13

**Authors:** Takemichi Fukasawa, Asako Yoshizaki-Ogawa, Ayumi Yoshizaki, Shinichi Sato

**Affiliations:** Department of Dermatology, The University of Tokyo Graduate School of Medicine, Tokyo, Japan; Department of Clinical Cannabinoid Research, The University of Tokyo Graduate School of Medicine, Tokyo, Japan; Department of Dermatology, The University of Tokyo Graduate School of Medicine, Tokyo, Japan; Department of Dermatology, The University of Tokyo Graduate School of Medicine, Tokyo, Japan; Department of Clinical Cannabinoid Research, The University of Tokyo Graduate School of Medicine, Tokyo, Japan; Systemic Sclerosis Center, The University of Tokyo Hospital, Tokyo, Japan; Department of Dermatology, The University of Tokyo Graduate School of Medicine, Tokyo, Japan; Systemic Sclerosis Center, The University of Tokyo Hospital, Tokyo, Japan

Rheumatology key message

- Guselkumab ameliorated the three pathological states of systemic sclerosis: immune abnormalities, fibrosis, and vascular damage.

Dear Editor, SSc is a systemic fibrosis disorder [1], in which autoimmunity plays an important role. B lymphocytes have diverse functions, such as producing antibodies, presenting antigens, and secreting cytokines. For example, B cells produce IL-6 or IL-23 (pro-inflammatory cytokines) and IL-10 or IL-35 (anti-inflammatory cytokines). B cell populations producing these cytokines are termed as (i) effector and (ii) regulatory B cells, respectively, and they have the function of (i) inducing or promoting, or (ii) inhibiting inflammation, through differentiating or activating other immune cells, including helper T (Th) cells [2]. IL-23 is produced by B cells, as well as dendritic cells and macrophages. Here, we treated three cases of psoriasis vulgaris (PsV) complicated by SSc with guselkumab, an IL-23 inhibitor that specifically binds to the IL-23 p19 subunit, and found a good therapeutic effect on both PsV and SSc. We also examined the effect of guselkumab on each symptom of SSc: immune abnormalities, fibrosis, and vasculopathy.

All cases were referred to us for close examination and treatment of suspected PsV. The close examination, including skin biopsy, confirmed the diagnosis of PsV [3]. In addition, these patients had skin sclerosis extending from the fingers to the upper arm. All cases were diagnosed with SSc based on the ACR/EULAR criteria [1]. Full methods are available in Supplementary Data S1, available at *Rheumatology* online. All patients have gastroesophageal reflux disease (GERD) by gastrointestinal endoscopy. One patient who was anti-topo I antibody positive (Case #2; Supplementary Table S1 available at *Rheumatology* online) had mild interstitial lung disease (ILD) on high-resolution CT, with percentage predicted forced vital capacity (FVC) and diffusion capacity of the lung for carbon monoxide (DLco) of 74.7% and 88.1%, respectively. In case #1, anti-RNA polymerase III antibody was positive and ACA was positive for case #3. After these systemic assessments, all patients refused oral treatment and preferred to be treated with biologics for PsV. For this purpose, 100 mg of s.c. guselkumab was administered at weeks 0 and 4, and at 8-week intervals thereafter, with no prior or other concomitant systemic therapy. Patient background information and clinical data at the start of treatment with guselkumab are shown in Supplementary Table S1.

In all cases, guselkumab resulted in a psoriasis area and severity index (PASI) [3] of 0 after 6 months of treatment (Fig. 1A). Skin sclerosis was also improved, with a decrease in modified Rodnan total skin thickness score (MRTSS) of >6 in each case (Fig. 1A). The combined response index for SSc (CRISS) [4] improved in all cases (Fig. 1A). Furthermore, GERD also improved after guselkumab administration, and the F scale, a measure of severity [5], decreased by >5 points (Fig. 1A). There were no exacerbations of ILD (Fig. 1A). No adverse events were observed. The laboratory findings remained unchanged (Fig. 1B). There was a significant increase in the number of capillaries and dilated capillaries. Giant capillaries and nailfold bleeding tended to increase, while the number of abnormal capillaries tended to decrease. Capillary flow velocity was significantly increased (Fig. 1C). Scleroderma capillary patterns showed an improving trend (Fig. 1D). T cells, Th17/Treg ratio, and IgG^+^ switched memory B cells in peripheral blood mononuclear cells were significantly decreased (Fig. 1E). Treg cells showed an increasing trend, while Th2 and Th17 cells showed a decreasing trend 6 months after treatment. Switched memory B cells, naïve B cells, plasma cells, B cells, and plasmablasts each showed a decreasing trend. These findings suggest that guselkumab may also act on T and B cells in the peripheral blood and ameliorate immune abnormalities in SSc.

**Figure 1.**
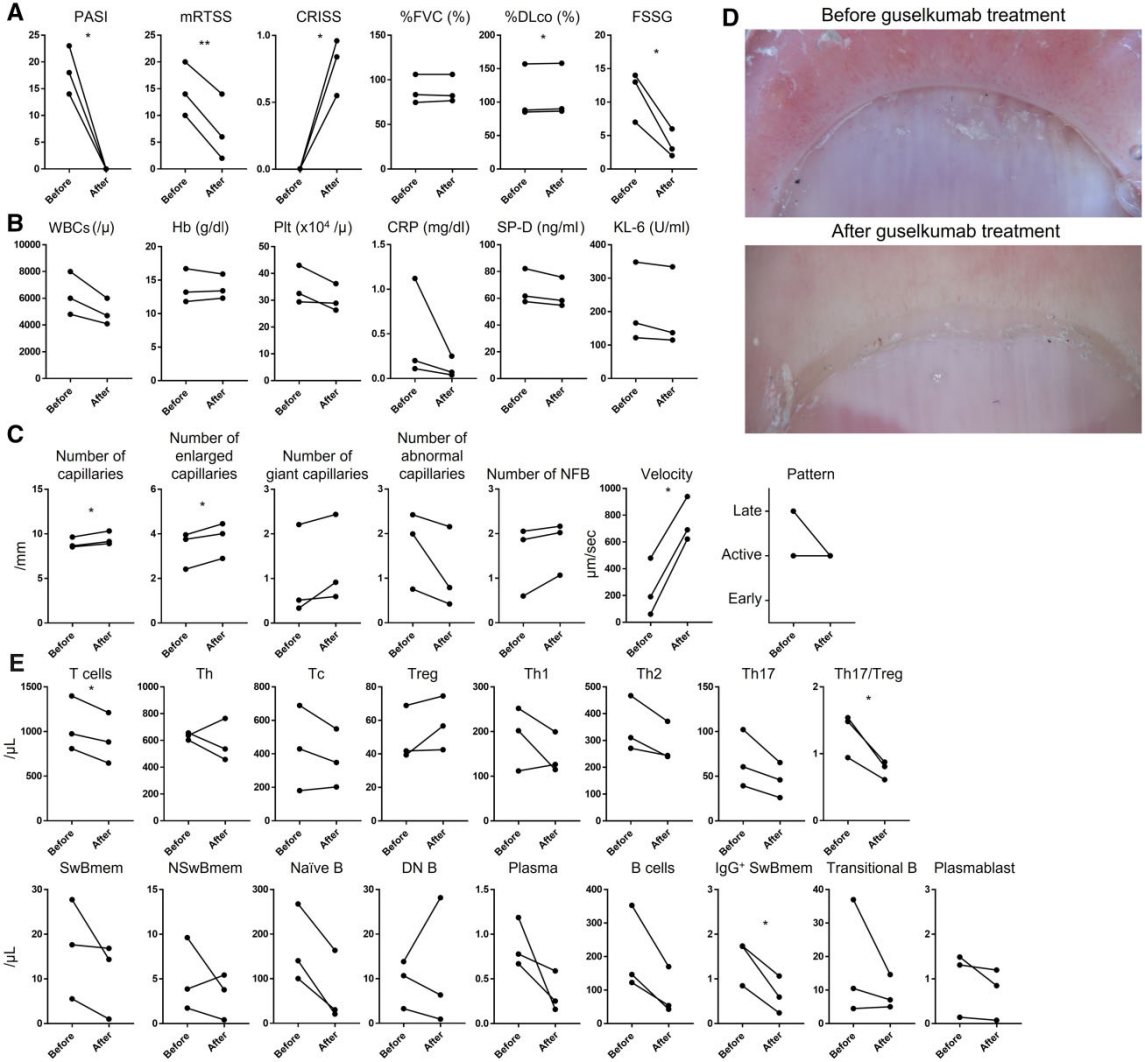
Changes in clinical findings, laboratory findings, and capillary abnormalities, and the profile of T and B cell subset analysis before and after guselkumab treatment. Changes in clinical findings (A), laboratory findings (B), and capillary abnormalities (C) before and after guselkumab treatment and their representative photographs (D). The transition of each T and B cell subset before and after guselkumab treatment (E). Spots represent each patient. Lines connecting dots represent changes in the same patient. PASI: psoriasis area and severity index; mRTSS: modified Rodnan total skin thickness score; CRISS: combined response index for SSc; %FVC: percentage predicted forced vital capacity; %DLco: percentage diffusion capacity of the lung for carbon monoxide; FSSG: Frequency Scale for the Symptoms of GERD; WBCs: white blood cells; Hb: haemoglobin; PLT: platelets; SP-D: surfactant protein D; KL-6: Krevs von den Lungen-6; NFB: nailfold bleeding; Th: helper T cells; Tc: cytotoxic T cells; Th1, type 1 helper T cells; Th2,:type 2 helper T cells; Th17: type 17 helper T cells; SwBmem: switched memory B cells; NSwBmem: non-switched memory B cells; DN B: double negative B cells. The combination of each cell surface marker is as follows; T cells: CD3^+^; CD4^+^ T cells: CD3^+^CD4^+^; CD8^+^ T cells: CD3^+^CD8^+^; Treg: CD3^+^CD4^+^CD25^+^CD127^+^; Th17: CD3^+^CD4^+^CCR6^+^CXCR3^-^; Th1: CD3^+^CD4^+^CCR6^-^CXCR3^+^; Th2: CD3^+^CD4^+^CCR6^-^CXCR3^–^; B cells: CD19^+^; switched memory B cells: CD19^+^IgD^-^CD27^+^; non-switched memory B cells: CD19^+^IgD^+^CD27^+^; naïve B cells: CD19^+^IgD^+^CD27^–^; DN B cells: CD19^+^IgD^–^CD27^–^; IgG^+^ class switched memory B cells: CD19^+^CD27^+^IgG^+^; plasmablast: CD19^+^CD20^–^CD38^+^; plasma cells: CD19^+^CD38^+^CD138^+^; transitional B cells: CD19^+^CD24^+^CD38^+^

IL-23 is a cytokine involved in the differentiation and maintenance of Th17 cells [6]. Th17 cells produce IL-17, a key cytokine in autoimmune inflammatory diseases such as psoriasis and collagen disease [7]. Th17 cells produce inflammatory cytokines such as IL-6 and IL-17, which act on endothelial cells or fibroblasts to induce vascular injury or fibrosis. Thus, biologics against IL-23 or IL-17 may be effective against autoimmune diseases, including SSc. For IL-17, clinical trials of an anti-IL-17 receptor A antibody are underway in Japan (NCT03957681) and have shown good results [8]. Currently, however, the efficacy of anti-IL-23 antibody therapy in SSc patients remains unknown because of a lack of clinical trials. The results of this study suggest that guselkumab may improve all three components of SSc: immune abnormalities, fibrosis, and vasculopathy.

The limitation of this study is that the number of patients is small and all patients are Japanese. A clinical trial with guselkumab is currently underway for SSc with moderate to severe skin sclerosis (NCT02207231). This report shows promising results and guselkumab will be a great gospel for patients. The present study complies with the Declaration of Helsinki. The whole study was approved by the ethics committee of the University of Tokyo Graduate School of Medicine. Written informed consent was obtained from all participants.

## Supplementary Material

kead287_Supplementary_Data

## Data Availability

The datasets generated and/or analysed during the current study are not publicly available but are available from the corresponding author upon reasonable request.
