# Supplementary material for: Impact of guselkumab on three cases of SSc accompanying psoriasis
Source: Rheumatology (Oxford). 2023 Jun 13;63(1):e6–8. doi: 10.1093/rheumatology/kead287 (PMC10765147; doi:10.1093/rheumatology/kead287)
Supplement: kead287_Supplementary_Data [file kead287_supplementary_data.docx]

**Impact of guselkumab on three cases of SSc accompanying psoriasis**

Takemichi Fukasawa^1,2^, Asako Yoshizaki-Ogawa^1^, Ayumi Yoshizaki^1,2,3*^, and Shinichi Sato^1,3^

^1^Department of Dermatology, The University of Tokyo Graduate School of Medicine, Tokyo, Japan.

^2^Department of Clinical Cannabinoid Research, The University of Tokyo Graduate School of Medicine, Tokyo, Japan.

^3^Systemic Sclerosis Center, The University of Tokyo Hospital, Tokyo, Japan.

*Address for correspondence and reprints requests: Dr. Ayumi Yoshizaki

Department of Dermatology, The University of Tokyo Graduate School of Medicine, 7-3-1 Hongo, Bunkyo-ku, 113-8655, Tokyo.

E-mail: [ayuyoshi@me.com](mailto:ayuyoshi@me.commiyoshizaki2022@gmail.com)

**Supplementary Data S1. Materials and Methods**

*Patients.*

  All samples were obtained from these three SSc patients, all of whom provided their signed informed consent for the study. All patients fulfilled the American College of Rheumatology classification criteria for SSc [1]. All patients had diffuse cutaneous SSc, according to the disease classification system proposed by LeRoy et al [2]. None of the SSc patients were being treated with oral corticosteroids, D-penicillamine, any other immunosuppressive therapy, gastrointestinal drugs, antifibrotic drugs, vasodilators, antihypertensive drugs, or topical treatments at the time of evaluation. All studies were approved by the Committee on Ethics of the University of Tokyo Graduate School of Medicine.

*Clinical assessment of patients.*

    A complete review of the medical history, physical examinations, and laboratory tests, including pulmonary function test were conducted for all patients. The severity of skin fibrosis was rated using the mRTSS; scale of 0-3, where 0 = normal, 1 = mild thickening, 2 = moderate thickening, and 3 = severe thickening, with a maximum possible score of 51, as previously described [3]. Organ involvement was defined in a manner as previously described [4]: for the lungs, interstitial lung disease is defined as bibasilar fibrosis on chest radiography and high-resolution computed tomography.

*Purification and stimulation of immune cells.*

    Heparinized blood samples were obtained from SSc patients. Antibodies used in this study described below; fluorescein isothiocyanate (FITC)-conjugated anti-human CD3 (7D6), CD24 (eBioSN3 (SN3 A5-2H10)), and CD25 (CD25-4E3) antibodies, phycoerythrin (PE)-Cy7-conjugated anti-human CD19 (HIB19), CD127 (eBioRDR5), and CD196 (CCR6) (R6H1) antibodies, allophycocyanin (APC)-conjugated anti-human CD8a (SK1), and CD20 (2H7) antibodies, PE-conjugated anti-human CD4 (S3.5), CD27 (O323), and CD38 (HB7) antibodies (all from Thermo Fisher Scientific), FITC-conjugated anti-human IgD (IA6-2), APC-conjugated anti-human IgG Fc (HP6017), CD3 (7D6), and CD138 (DL-101) antibodies (all from Biolegend), VioBright FITC-conjugated anti-human CD183 (CXCR3) antibodies (REA232, Miltenyi Biotec). All samples were subjected to Fc blocking with 5 μg of human IgG (polyclonal human IgG, Sigma, Cat. No. I-4506) for 5-10 minutes. The samples were then stained, washed, and flowed into a flowcytometry for measurement.

*Observation of the nailfold capillaries.*

We examined the nailfolds of all fingers for capillaroscopic changes. The number of NVC findings in each finger and their distribution was recorded. Dermatoscopy of the nailfolds was performed in all patients using a Derma9500 digital dermatoscope (Derma Medical Inc., Japan) and photos were obtained by attaching the dermatoscope to a Canon Powershot G12 (Canon, Japan) digital camera. NVC was performed by TOKU Capillaro-01 (Toku Co., Tokyo, Japan) which also enabled to take digital photos of the examined nailfold simultaneously. The patients were examined with NVC for the determination of their NVC findings. They were asked to refrain from caffeine for 12 hours before the test. Patients were positioned in a supine position for 15 minutes at room temperature (22 to 25°C). For each image, capillaroscopic parameters including nailfold bleeding (NFB) and irregularly enlarged capillaries were evaluated by the same dermatologist. According to the previous definition [5,6], the following quantitative capillaroscopic variations were evaluated: number of distal capillaries per mm (density); number of enlarged loops per mm (irregular or homogeneous increase of capillary diameter ≧20 μm); number of distal abnormal capillaries per mm (not hairpin shape, once or twice crossing limbs, tortuous shape, and convex head); microhemorrhages per mm (presence of 1 or more dark red masses characterized by hemosiderin deposits due to capillary injury or thrombosis). Each parameter was averaged for 10 fingers and was considered the patient's value. The average velocity of the capillaries (µm/sec) was measured for 10 fingers on each side and averaged for 10 fingers to determine the patient's average velocity. The diameter of normal blood vessels near the center of the nail cage was measured. For qualitative evaluation, Cutolo's classification [7] was used: early pattern, defined as no loss of density and few giant capillaries; active pattern, defined as moderate vascular loss, increased giant capillaries, preserved or mildly abnormal morphology, and heavy bleeding; late pattern, defined as extreme loss of capillaries, loss of giant capillaries, stray normal capillary structures, and absence of hemorrhage points. These findings were analyzed using capillary anemometer CAM, the CapiScope software, and the Capimetrics software (KK Technology, Axminster, UK). Capillary assessment was performed by two blinded assessors (TF, AY).

*Statistical analysis*

Statistical significance was tested by applying Mann-Whitney U-test or paired student t-test. All statistical analyses were performed with the use of Graphpad Prism 7. A p-value < 0.05 was considered statistically significant.

**Supplementary References**

1. Masi, A.T. Preliminary Criteria for the Classification of Systemic Sclerosis (Scleroderma). Subcommittee for Scleroderma Criteria of the American Rheumatism Association Diagnostic and Therapeutic Criteria Committee. *Arthritis Rheum.* **1980**, *23*, 581–590, doi:10.1002/ART.1780230510.

2. LeRoy, E.; Black, C.; Fleischmajer, R.; Jablonska, S.; Krieg, T.; Medsger, T.J.; Rowell, N.; Wollheim, F. Scleroderma (Systemic Sclerosis): Classification, Subsets and Pathogenesis. *J Rheumatol.* **1988**, *15*, 202–205.

3. Clements, P.J.; Lachenbruch, P.A.; Seibold, J.R.; Zee, B.; Steen, V.D.; Brennan, P.; Silman, A.J.; Allegar, N.; Varga, J.; Massa, M.; et al. Skin Thickness Score in Systemic Sclerosis: An Assessment of Interobserver Variability in 3 Independent Studies. *J. Rheumatol.* **1993**, *20*, 1892–1896.

4. Sato, S.; Ihn, H.; Kikuchi, K.; Takehara, K. Antihistone Antibodies in Systemic Sclerosis. *Arthritis Rheum.* **1994**, *37*, 391–394, doi:10.1002/art.1780370313.

5. Sebastiani, M.; Manfredi, A.; Cassone, G.; Giuggioli, D.; Ghizzoni, C.; Ferri, C. Measuring Microangiopathy Abnormalities in Systemic Sclerosis Patients: The Role of Capillaroscopy-Based Scoring Models. *Am. J. Med. Sci.* **2014**, *348*, 331–336, doi:10.1097/MAJ.0000000000000282.

6. Smith, V.; Beeckman, S.; Herrick, A.L.; Decuman, S.; Deschepper, E.; De Keyser, F.; Distler, O.; Foeldvari, I.; Ingegnoli, F.; Müller-Ladner, U.; et al. An EULAR Study Group Pilot Study on Reliability of Simple Capillaroscopic Definitions to Describe Capillary Morphology in Rheumatic Diseases. *Rheumatology (Oxford).* **2016**, *55*, 883–890, doi:10.1093/RHEUMATOLOGY/KEV441.

7. Cutolo, M.; Sulli, A.; Smith, V. Assessing Microvascular Changes in Systemic Sclerosis Diagnosis and Management. *Nat. Rev. Rheumatol.* **2010**, *6*, 578–587, doi:10.1038/nrrheum.2010.104.

**Supplementary Table S1. The background and clinical data of patients.**

|  | Case #1 | Case #2 | Case #3 |
| --- | --- | --- | --- |
| Age (years) | 67 | 68 | 78 |
| Sex | Male | Female | Male |
| Disease duration (months) |  |  |  |
| PsV | 17 | 24 | 12 |
| SSc | 10 | 15 | 6 |
| Type of SSc | dcSSc | dcSSc | dcSSc |
| Autoantibody profiles | RNAP | Topo I | CENP |
| PASI | 18 | 23 | 14 |
| MRTSS | 20 | 14 | 10 |
| CRISS | 0.004 | 0.004 | 0.004 |
| ILD | - | + | - |
| %FVC (%) | 83.4 | 74.7 | 106 |
| %DLco (%) | 85.2 | 88.1 | 157 |
| GERD | + | + | + |
| F-scale | 14 | 13 | 7 |
| Raynaud’s phenomenon | + | + | + |
| Skin ulcers | - | - | - |
| Arthritis | - | - | - |
| Pulmonary hypertension | - | - | - |
| Laboratory findings |  |  |  |
| WBC (/μL) | 8000 | 6000 | 4800 |
| Hb (g/dL) | 16.7 | 11.8 | 13.2 |
| PLT (x10^4^/μL) | 43.0 | 32.5 | 29.4 |
| CRP (mg/dL) | 1.12 | 0.20 | 0.11 |
| SP-D (ng/mL) | 82.2 | 57.5 | 61.7 |
| KL-6 (U/mL) | 122 | 348 | 166 |

dcSSc, diffuse cutaneous SSc; lcSSc, limited cutaneous SSc; WBC, white blood cells; Hb, hemoglobin; PLT, platelets; CRP, C-reactive protein; SP-D, surfactant protein D; KL-6, Krevs von den Lungen-6; RNAP, anti-RNA polymerase III antibody; Topo I, anti-topoisomerase I antibody; CENP, anti-centromere protein antibody; +, presence; -, absence.
